# TGF-β and the Tissue Microenvironment: Relevance in Fibrosis and Cancer

**DOI:** 10.3390/ijms19051294

**Published:** 2018-04-26

**Authors:** Laia Caja, Francesco Dituri, Serena Mancarella, Daniel Caballero-Diaz, Aristidis Moustakas, Gianluigi Giannelli, Isabel Fabregat

**Affiliations:** 1 Science for Life Laboratory, Department of Medical Biochemistry and Microbiology, Biomedical Center, Uppsala University, Box 582, 75123 Uppsala, Sweden; aris.moustakas@imbim.uu.se; 2 National Institute of Gastroenterology, “S. de Bellis” Research Hospital, Castellana Grotte, 70013 Bari, Italy; francesco.dituri@irccsdebellis.it (F.D.); mancarella.serena@gmail.com (S.M.); gianluigi.giannelli@irccsdebellis.it (G.G.); 3 TGF-β and Cancer Group, Oncobell Program, Bellvitge Biomedical Research Institute (IDIBELL), Gran Via de l’Hospitalet, 199, 08908 Barcelona, Spain; dcaballero@idibell.cat; 4 Oncology Program, CIBEREHD, National Biomedical Research Institute on Liver and Gastrointestinal Diseases, Instituto de Salud Carlos III, 28029 Madrid, Spain; 5 Department of Physiological Sciences, Faculty of Medicine and Health Sciences, University of Barcelona, L’Hospitalet, 08907 Barcelona, Spain

**Keywords:** TGF-β, fibrosis, cancer, HCC, microenvironment, CAF, hepatic stellate cells, extracellular matrix, galunisertib

## Abstract

Transforming growth factor-β (TGF-β) is a cytokine essential for the induction of the fibrotic response and for the activation of the cancer stroma. Strong evidence suggests that a strong cross-talk exists among TGF-β and the tissue extracellular matrix components. TGF-β is stored in the matrix as part of a large latent complex bound to the latent TGF-β binding protein (LTBP) and matrix binding of latent TGF-β complexes, which is required for an adequate TGF-β function. Once TGF-β is activated, it regulates extracellular matrix remodelling and promotes a fibroblast to myofibroblast transition, which is essential in fibrotic processes. This cytokine also acts on other cell types present in the fibrotic and tumour microenvironment, such as epithelial, endothelial cells or macrophages and it contributes to the cancer-associated fibroblast (CAF) phenotype. Furthermore, TGF-β exerts anti-tumour activity by inhibiting the host tumour immunosurveillance. Aim of this review is to update how TGF-β and the tissue microenvironment cooperate to promote the pleiotropic actions that regulate cell responses of different cell types, essential for the development of fibrosis and tumour progression. We discuss recent evidences suggesting the use of TGF-β chemical inhibitors as a new line of defence against fibrotic disorders or cancer.

## 1. Introduction: Transforming Growth Factor-β (TGF-β), Fibrosis and Cancer

After tissue injury, due to infection, physical trauma or exposure to toxic substance the damaged tissue initiates reparative processes to restore the organ structure and function. Any of the original tissue damage inducers can lead to abnormal and excessive accumulation of extracellular matrix (ECM) constituents, particularly collagens, triggering the development of fibrosis. 

Fibrosis occurs in several organs. One of the most common forms of fibrosis is idiopathic pulmonary disease that affects the lung. Diabetes, hypertension and chronic glomerulonephritis can cause chronic kidney fibrotic disease. Several liver diseases lead to hepatic fibrosis: alcoholic steatohepatitis, non-alcoholic steatohepatitis and non-alcoholic fatty-acid liver disease, virally induced hepatic fibrosis and primary biliary cirrhosis. Nearly all aetiologies of heart disease can lead to excessive deposition of ECM by cardiac fibroblasts resulting in heart failure. The fibrotic response also contributes to the progression of diseases not commonly described as scarring, such as small-airway remodelling in chronic pulmonary disease and in vascular remodelling in pulmonary hypertension and atherosclerosis. Fibrosis leads to progressive loss of tissue function and eventually to organ failure; it has been estimated to contribute to approximately 45% of deaths in the developed world [1,2]. 

The fibrotic process, independently of the organ where it occurs, is characterized by inflammation, altered epithelial-mesenchymal interactions and proliferation of fibroblasts. One of the hallmarks of fibrosis is the differentiation of fibroblasts into myofibroblasts, which are contractile cells that express α-smooth muscle actin (α-SMA), exhibiting hyperproliferation and dysregulated metabolism of ECM. The infiltration of immune cells into the fibrotic tissue also plays a key role in amplifying the fibrotic response by secreting several cytokines and chemokines responsible for the differentiation of myofibroblasts, stimulation of ECM deposition and further recruitment of immune cells (macrophages, T cells) [3,4].

Desmoplasia or accumulation of ECM, angiogenesis, inflammation and the suppression of anti-tumorigenic adaptive immune cell responses are also characteristics of the tumour microenvironment that promote tumour growth [5,6]. As in the fibrotic tissue, in the tumour milieu there is an activation of resident and infiltrating fibroblasts, the cancer-associated fibroblasts (CAFs), which produce excessive deposition of ECM, providing a scaffold for the infiltration of other cells (such as immune cells) and a substrate for cell migration. The incoming innate immune cells shift towards a pro-tumorigenic phenotype in response to cytokines and chemokines released by the tumour cells, CAFs and the immune cells themselves [7,8]. 

Transforming growth factor-β (TGF-β) is a cytokine essential for the induction of the fibrotic response and for the activation of the cancer stroma. TGF-β can control several functions exerted by most of the cells involved in the fibrotic tissue and in the tumour microenvironment, promoting myofibroblast differentiation and the recruitment of immune cells, inhibiting the anti-tumour immune responses and affecting epithelial and endothelial cell differentiation [7,9,10]. Even though TGF-β can induce such different responses in different cell types, its signalling pathway can be simplified as TGF-β binding to its TGF-β Receptor II (TβRII), which promotes the interaction of the TβRII dimer with two TβRI molecules, enabling TβRII to phosphorylate the TβRI on serine and threonine and thus activating TβRI catalytic activity. The catalytically active TβRI phosphorylates the C-terminal serine residues of receptor-activated (R-) Smads, which in the case of TGF-β are Smad2 and Smad3. Once phosphorylated, R-Smads oligomerize with Smad4 forming trimeric protein complexes. These complexes are translocated to the nucleus where they associate with other transcription factors in order to induce or repress gene expression. TGF-β also induces non-Smad pathways, including mitogen-activated protein kinases (MAPK), phosphoinositide-3-kinase (PI3K), PP2A phosphatase and Rho GTPases (Rho). These non-Smad signals play different roles: they are involved in TGF-β-mediated biological responses but they can also regulate the canonical Smad pathway [11,12,13,14].

In this review, we summarize how TGF-β and the tissue microenvironment cooperate to promote fibrosis and tumour progression, through pleiotropic actions that regulate cell responses of different cell types: epithelial cells, endothelial cells, fibroblasts and immune cells. 

## 2. Stromal Activation of Latent TGF-β: Role of Integrins

TGF-β is a highly pleiotropic cytokine and its synthesis and activation must be tightly controlled. TGF-β is stored in the matrix as part of a large latent complex bound to the latent TGF-β binding protein (LTBP) and matrix binding of latent TGF-β complexes, which is required for an adequate TGF-β function [15]. Sequestration of latent TGF-β in the ECM was demonstrated to be crucial for the proper activation of the latent cytokine. There are four LTBPs; LTBP-1 was initially identified by its capacity to form latent complexes with TGF-β by binding the TGF-β pro-form LAP (Latency Associated Protein) through disulphide bonds in the endoplasmic reticulum [16]. Although in the trans Golgi covalent junctions in the pro-TGF-β precursor are disrupted by proteolytic cleavage, LAP and TGF-β remain strongly bound by means of non-covalent interactions and LAP-TGF-β-LTBP form the large latent complex (LLC) which is secreted. LTBPs -1, -3 and -4 are critical for correct deposition and subsequent bioavailability of TGF-β in the ECM, whereas LTBP2 has TGF-β independent functions.

Initial studies suggested an essential role for LTBPs in targeting the LLC to the ECM. Nowadays, strong evidence suggests that fibrillin (Fbn) and fibronectin (FN) are the main matrix proteins responsible for the association of LTBPs with the ECM. In the ascending aortas and lungs of *Fbn1*^−/−^ mice, LTBP-3 and LTBP-4 are not incorporated into microfibrils, whereas LTBP-1 is still deposited. Cells deficient for both fibrillin-1 and fibrillin-2 still incorporate LTBP-1 in their matrix. However, blocking the FN network with functional upstream domain (FUD) peptide, which blocks FN fibre assembly, in *Fbn1*^−/−^ cells completely abrogated the deposition of LTBP-1 [17,18]. Together, these data indicate that LTBP-3 and LTBP-4 associate with the matrix on fibrillin-1 microfibrils; in contrast, LTBP-1 association might depend on a FN network. However, recent studies have also indicated that LTBP-1 has the capacity to form autonomous oligomeric structures, independent of other matrix components, in a calcium-dependent manner and enhanced by heparin sulphate. In this way, LTBP-2 may directly contribute to matrix architecture and potentially allow fibrillin interactions providing tethering for TGF-β activation [19].

While associated to the matrix in form of LLC, the TGF-β remains inert. Indeed, various biological processes that are dependent on TGF-β signalling are controlled in the tissues by the activation of latent TGF-β, which will allow the exposure or release of the cytokine to bind its specific TGF-β receptors in the cells. TGF-β stored in the matrix can be activated by the cell contractile force, which is transmitted by integrins [20,21]. Matrix straining and stiffening lower the threshold for TGF-β activation by increasing the mechanical resistance to cell pulling [22,23,24]. αv Integrins are the major regulators of the local activation of latent TGF-β, which requires the binding of αv integrin to an RGD sequence in the prodomain and the exertion of force in this domain [20]. Mice with a mutation in *Tgfb1* encoding a non-functional variant of the RGD sequence display the major features of *TGFb1^−^*^/*−*^ mice [25]. It has been proposed that forces acting on elastic fibres would extend fibrillins and LTBPs and this could weaken their association with TGF-β family members, enabling release and activation [26]. Elucidation of the basis for ligand binding specificity by the integrin β subunit has revealed the contribution of three different domain loops, whose understanding will allow advances in the comprehension about how β-subunits contribute to integrin-ligand specificity and the rationale for the design of potential antagonists [27].

Since activation of the latent form of TGF-β is required for releasing its active form, different elements of this mechanism, including specific integrins and matrix protein interactions, may be pharmacologically targeted in those pathologies where TGF-β plays a role, such as fibrosis and cancer. An elegant study by Henderson et al. [28] demonstrated that deleting αv integrin in hepatic stellate cells (HSC)—the main drivers of fibrogenesis in the liver—protected mice from CCl_4_-induced hepatic fibrosis. Furthermore, pharmacological blockade of αv integrins attenuated both liver and lung fibrosis, even when the drug was administered after fibrosis was established. A recent study indicates that integrin αvβ6 is expressed in hepatic progenitor cells and is required for the progenitor cell response in mouse models of chronic biliary injury [29]. Selective pharmacologic antibody targeting αvβ6 inhibited progenitor expansion, a process that was rescued by addition of bioactive TGF-β and provided in vivo protection from liver fibrosis and tumorigenesis. An alternative approach would be inhibiting the binding of latent TGF-β to FN fibrils, via a monoclonal antibody targeting the growth factor binding domain of FN; the utility of this approach could be tested genetically through use of a FN deletion mutant that cannot associate with latent TGF-β [30]. This procedure has been effective in disrupting epithelial-mesenchymal transition (EMT), indicating a crucial role for FN in EMT in which the assembly of FN fibrils serves to localize TGF-β signalling to drive this process. This may be a strategy that allows for global blockage of disease progression in pathologies associated with EMT, such as fibrosis and cancer.

## 3. TGF-β as a Master Regulator of Extracellular Matrix Remodelling

TGF-β is considered a critical player in chronic fibrosis of many organs, including lung, kidney, liver or skin. In fact, up-regulation of the expression and synthesis of the major ECM proteins FN and collagen (COL), was one of the earliest proposed roles for TGF-β. Dr. Massagué’s lab first demonstrated that the relative incorporation of FN and COL into the matrix increases in response to TGF-β [31], which also regulates the expression of cell adhesion protein receptors, such as integrins [31,32] and metalloprotease inhibitors, such as tissue inhibitors of metalloproteinases (TIMP) [33]. Simultaneous expression of TGF-β and ECM proteins during experimental models of liver fibrosis led Thorgeirsson’s group to propose the possibility that TGF-β plays an important role in the development of fibrosis [34].

We now know that proteins up-regulated by TGF-β also include basement membrane proteins, such as laminin and many other ECM proteins, such as osteopontin, tenascin, elastin, decorin and more. We also know that TGF-β induces the conversion of fibroblasts (or HSC in the liver) into myofibroblasts, a process mediated by the activation of the Nicotinamide adenine dinucleotide phosphate (NADPH) oxidase 4 (NOX4) [35,36]. Myofibroblasts later contribute to further distorting the ECM by secreting different ECM proteins as well as matrix metallopoteinases (MMPs), the main extracellular matrix enzyme family that degrades collagen. Additionally, myofibroblast can proliferate within the ECM. All these events change the ECM structure during fibrosis [37,38,39]. Recent evidence also involves TGF-β in the differentiation of mesenchymal stem cells (MSC) into myofibroblasts. TGF-β activates RhoA/Rho-associated protein kinase 1 (ROCK) signalling functions, which act as a molecular switch regarding the fate of MSCs in arterial repair/remodelling after injury [40]. TGF-β mediates phenotypic changes affecting contractile proteins and collagen I in vascular smooth muscle cells (VSCM), leading to greater cellular and extracellular matrix stiffness. These alterations may contribute to the known aortic rigidity that precedes or accompanies aneurysm formation in patients with Marfan’s syndrome [41]. Additionally, TGF-β activates myofibroblasts and other stromal cells to enhance the synthesis of collagen cross-linking proteins, such as the lysyl oxidase (LOX) family of matrix-remodelling enzymes, which increase the rigidity of the collagen network. TGF-β dependent expression of lysyl oxidase-like (LOXL) 4 plays a role in vascular ECM homeostasis, contributing to vascular processes associated with ECM remodelling and fibrosis [42]. TGF-β-induced up-regulation of LOXL2 in tumours, such as hepatocellular carcinoma (HCC), is critical for remodelling ECM components in the tumour microenvironment and metastatic niche [43]. Finally, recent evidence indicates that TGF-β, through increased FN trafficking, is a direct and rapid inducer of the fibrillogenesis required for TGF-β-induced cell migration. In response to TGF-β, cell-surface-internalized FN is not degraded by the lysosome but instead is recycled for fibrillogenesis, a process dependent of the TβRII [44].

## 4. TGF-β Signalling in Fibrosis and Cancer-Related Invasiveness

The specific signalling pathways activated by TGF-β in various cell types during tissue fibrosis and cancer progression are diverse and provide evidence for a complex network of signalling molecules that play important biological roles under different pathophysiological conditions. Covering the majority of such signalling mechanisms exceeds the capacity of a single article; however, we here provide some illustrative examples by focusing mostly on fibrotic conditions and selectively providing mechanisms that drive tumour cell invasiveness. As explained above, TGF-β signalling involves activation of Smad proteins and various protein and lipid kinases and the summary outlined below clearly demonstrates the importance of cooperation between Smad and non-Smad pathways in mediating pathophysiological responses during disease progression.

ECM induction, the hallmark of fibrotic responses, involves for example collagen type I (COL1A2) synthesis, which is transcriptionally induced by Smad3 and the co-activator p300 in dermal fibroblasts responding to TGF-β [45]. The tumour suppressor p53 protein counteracts the Smad3-dependent activity in such fibroblasts and thus indirectly protects from fibrotic development [45]. Similarly, in a mouse model of ureteral obstructive fibrosis, the cooperation of Smad signalling together with Src and Mitogen-activated protein kinase (MAPK) signalling induces expression of plasminogen activator inhibitor 1 (PAI-1), an important extracellular marker of kidney fibrosis [46]. Additional ECM components, including connective tissue growth (CTGF) and MMP2 are regulated by Smad2 or Smad3 in lung fibrosis [47]. Selective interference with Smad2 or Smad3 using short interfering RNAs indicated that CTGF induction (and coordinate E-cadherin repression) required Smad3 activity, whereas MMP2 induction required Smad2 signalling in lung epithelial cells undergoing EMT and fibrotic development in response to TGF-β1 [47]. In hepatic stellate cells, CTGF transcriptional induction requires both Smad3 and signalling by the Janus Kinase 1 (JAK1)-Stat3 pathway, which is activated by the TGF-β receptors via the MAP-kinase and PI3′-kinase signalling modules [48]. The kinase JAK1 can directly associate with the TβRI, a mechanism that deserves further biochemical dissection during liver fibrosis [49]. In kidney fibrotic models, the activity of Smad signalling is regulated by phosphorylation on tyrosine residues of the TβRII [50]; dephosphorylation of these tyrosines by the T cell protein tyrosine phosphatase can be activated by integrin α1β1 signalling, which counteracts Smad activation by the TGF-β receptors and counterbalances the onset of fibrosis [50]. In lung epithelial cells that are implicated in pulmonary fibrosis, TGF-β activates Smad3 and the focal adhesion kinase (FAK) to mediate EMT and fibrotic marker expression such as α-SMA [51]. As discussed more extensively later, liver fibrosis is a process that depends strongly on Smad3 activity; interestingly, liver fibrosis can be modulated by signalling activities taking place in endothelial cells of the liver vasculature, whereby, the ETS-related gene (ERG) transcription factor suppresses Smad3 function and protects from the onset of liver fibrosis [52]. The critical role of Smad3 in tissue fibrosis stimulated efforts to develop inhibitors of its activity. Accordingly, the compound HSc025 inhibits Smad3 activation and downstream gene regulation, including collagen and fibronectin, in vitro, whereas in vivo administration in mice could protect from bleomycin-induced lung fibrosis or from sclerotic skin development [53]. In a similar manner, small peptides derived from the endocytic protein sortin nexin 9 that can penetrate the cell membrane and which bind tightly to Smad3 and inhibit its transcriptional activity, provided effective anti-fibrotic action in vitro in lung fibroblasts and in mice that develop lung fibrosis [54]. These peptides could not inhibit Smad2 signalling in the same cell types.

Beyond direct Smad signalling, the TGF-β receptors activate various lipid and protein kinases. The E3 ubiquitin ligases TNF-α receptor activated factor (TRAF) 4 and 6 are key mediators of non-degradative TGF-β receptor poly-ubiquitination, which acts as a scaffold for the recruitment of various signalling proteins, including the MAP-kinase kinase kinase TGF-β-associated kinase 1 (TAK-1), which mediates downstream non-Smad signalling and contributes to cancer cell invasion [55,56]. TRAF6 can also poly-ubiquitinate the regulatory subunit p85 of the PI3′-kinase, thus leading to activation of this lipid kinase and further activation of the protein kinase B (Akt), also involved in the regulation of EMT and tumour cell invasiveness [57]. Activation of the PI3′-kinase/Akt signalling module in fibroblasts or in fibrotic kidney cells also involves the activity of the FAK or the small GTPase Rac1, respectively, mechanisms that require deeper investigation [14]. Is for example Rac1 GTPase and FAK activity modulated by TRAF6-dependent poly-ubiquitination? Interestingly, recent evidence on the role of the de-uibiquitinase USP2a that interacts with TβRI has pointed to the role of non-degradative ubiquitination of TβRI and TβRII for Smad2 and Smad3 recruitment to the receptors and phosphorylation in invasive cancer cells, thus linking even tighter the regulation of Smad with non-Smad pathways at the level of TGF-β receptors [58]. Beyond ubiquitin-mediated non-Smad signalling, the TβRI is known to phosphorylate tyrosine residues with relatively weaker potency, leading for example to activation of the ShcA adaptor protein after dual tyrosine and serine phosphorylation, leading to MAP-kinase/ERK activation in tumour cells [59]. In the context of fibrotic cells, such TGF-β-mediated MAP-kinase signalling can regulate the expression of ECM components such as fibronectin in kidney cells and CTGF in liver cells, via the p38 MAP-kinase [60,61].

Another major signalling node acting downstream of TGF-β under fibrotic conditions is the mammalian target of rapamycin (mTOR) kinase complex. Early studies pointed to the effectiveness of rapamycin in blocking pro-fibrotic actions of TGF-β in the liver [62]. In kidney fibrosis, TGF-β activates the c-Abl protein kinase together with mTOR complex 1 (mTORC1) in a Smad-independent manner to mediate ECM protein induction via fibroblast activation [63]. The second mTOR complex, mTORC2, can also mediate fibrotic ECM synthesis in kidney fibroblasts [64]. Accordingly and similar to liver fibrosis, rapamycin can be effective in blocking kidney fibrosis [65]. A similar signalling pathway involving the mTORC2 complex is important during EMT and cancer cell invasion [66] and operates during excessive ECM synthesis in fibrotic lungs [67]. 

As mentioned earlier, this section covers selective and illustrative signalling examples downstream of TGF-β. Trying to be as inclusive as possible, it is worth explaining that fibroblast to myofibroblast activation and α-SMA expression induced by TGF-β involves RhoA small GTPAse signalling, whereby TGF-β induces expression of the RhoA activator, the guanine exchange factor GEF-H1/Lfc, which promotes cell migration [68]. By inducing mitochondrial reactive oxygen species (ROS) in lung fibroblasts, TGF-β can contribute to lung fibrosis, a mechanism that implicates the NOX4 [69], as detailed further later. TGF-β also activates the sphingosine kinase pathway in activated myofibroblasts as Smad signalling regulates expression of this kinase, which regulates expression of sphingosine receptors, which act as important mediators of muscle fibrosis [70]. It is therefore apparent that development of fibrosis or cancer-associated invasiveness is actively promoted by TGF-β signalling, which involves multiple cross talking pathways, usually aiming at the regulation of ECM expression and remodelling.

## 5. Cancer-Associated Fibroblasts (CAFs): Their Origin and Links to the Epithelial-Mesenchymal Transition (EMT) in Fibrosis and Cancer

In fibrotic tissues, TGF-β, among other inflammatory cytokines, promotes a fibroblast to myofibroblast transition. Under normal tissue repair, a transient activation of myofibroblasts is necessary to allow the restoration of the tissue; however, persistent activation of myofibroblasts causes, as previously mentioned, accumulation of extracellular matrix leading to fibrosis. As recently reviewed in liver fibrosis [71], myofibroblasts can be originated from different sources, such as bone marrow-derived cells, mesothelial cells, HSCs and portal fibroblasts. Interestingly, epithelial cells have also been proposed to give rise to myofibroblasts in several fibrotic tissues such as kidney, lung and in the liver [72,73,74]. Here, we will mainly focus on the origin of myofibroblasts from epithelial cells. In order for epithelial cells to become myofibroblasts they need to undergo an EMT.

EMT plays a major role during embryonic development and during fibrosis and cancer. EMT takes place when epithelial cells lose their cuboidal shape, lose the expression of adherence and tight junction proteins, which leads to weak cell-cell contacts and reorganization of their actin cytoskeleton; while the cells acquire the expression of mesenchymal proteins (fibronectin, vimentin, *N*-cadherin), they adopt a fibroblast-like architecture favouring cell migration and invasion. EMT is induced by many growth factors, among them TGF-β being a very potent inducer, which regulate the expression and activity of several transcription factors known as EMT-TFs (Snail1/Snail, Snail2/Slug, ZEB1, ZEB2, Twist1/Twist and more) that are the responsible actors to execute the change in cell differentiation that is EMT [75]. The gene and protein markers used to identify the generation of mesenchymal cells after EMT in the context of fibrosis are FSP1 (Fibroblast-specific protein 1), α-SMA and collagen I along with vimentin and desmin, whose expression increases concomitant with a reduction in levels of expression of epithelial markers (E-cadherin and certain cytokeratins). Cells that co-express epithelial and mesenchymal markers represent an intermediate stage of EMT [76].

There are many in vitro studies that support the idea that TGF-β induces EMT in non-tumorigenic epithelial cells and transforms them into fibroblast-like cells. Different epithelial cell types in the lung have been shown to undergo EMT after TGF-β treatment; for example in alveolar epithelial cells [77] via FoxM1/Snail1 [78], or in pleural mesothelial cells [79]. In mammary epithelial cells TGF-β induces EMT via mTOR and PI3K [80,81,82]. In rat and human foetal hepatocytes, autocrine expression of TGF-β is linked with EMT and survival signalling pathways [83,84], where TGF-β induces Snail1 expression to promote EMT and to confer resistance to apoptosis [85]. In adult mouse liver hepatocytes TGF-β induces a fibroblast-like phenotype [86], while in adult rat hepatocytes it does not [87], which is an interesting observation worth exploring deeper.

There has been a serious effort to prove or refute the model whereby myofibroblasts originate from epithelial cells in vivo using cell fate mapping. In the kidney, it has been described that a sub-population of myofibroblasts has either epithelial or endothelial origin when fibrosis is induced by a unilateral ureteral obstruction (UUO), where these cells undergo an EMT or an Endothelial-Mesenchymal Transition (EndMT) process, respectively [72,88]. However, other fate mapping models have shown that epithelial cells do not contribute to the acquisition of a myofibroblast phenotype [89,90]. Interestingly, another study reported that expression of Snail in renal epithelial cells induces a partial EMT after UUO-induced kidney fibrosis, which is not sufficient for their delamination from the tubules to contribute to the myofibroblasts population; however, expression of Snail in the epithelial cells allows the production of TGF-β which can then act on resident fibroblasts and bone-marrow derived mesenchymal stem cells to promote their differentiation towards myofibroblasts [91]. Specific KO of Twist or Snail in tubular epithelial cells have allowed to prove that these EMT-TFs play a key role in kidney fibrosis induced by different methods. Lack of Snail or Twist resulted in reduced EMT concomitant with reduced immune infiltration; moreover, these models showed that the partial EMT that renal epithelial cells undergo under fibrotic conditions is responsible for the deregulated expression and functionality of several solute and solvent transporters in these cells as well as their arrest in the G2 phase [92]. In lung fibrosis, there are also contradictory data from fate mapping models regarding the epithelial origin of myofibroblasts. Some studies support the contribution of epithelial cells to the fibroblast population in lung fibrosis induced by overexpression of TGF-β or exposure to bleomycin [73,93,94]. However, there are also reports against EMT occurring in lung fibrosis [95]. Cell fate mapping has also challenged the contribution of epithelial cells in liver fibrosis: some authors claim that hepatocytes contribute to myofibroblast activation undergoing EMT after CCl_4_-induced fibrosis [96], while other studies reported that neither hepatocytes nor cholangiocytes undergo EMT in liver fibrosis [97,98]. However, Snail expression in hepatocytes has been proven to be necessary for CCl_4_-induced liver fibrosis, as its expression is required for immune infiltration [99]. Finally, in an intestinal fibrosis mouse model of Crohn’s disease the contribution of EMT in giving rise to fibroblasts has been reported [100]. In some of the models that support EMT occurring in vivo [73,94] the TGF-β ligand or its receptor, ALK5, are thought to be directly involved in the induction of EMT.

Similarly, in the tumour microenvironment resident fibroblasts, pericytes and bone marrow-derived mesenchymal cells, endothelial cells via EndMT or epithelial tumour cells can undergo an EMT process and become CAFs [101,102]. There have been a few reports proving the epithelial origin of CAFs: breast cancer cells from a patient biopsy have been characterized in vitro and have been found to co-express low levels of keratins together with high levels of α-SMA and these cells also exhibited a fibroblast-like morphology [103]. In a xenograft model of GFP-labelled human lung cancer cells, it has been reported that 15% of CAFs were of human origin, while the rest were murine, proving an epithelial origin of a subpopulation of the fibroblasts [104]. However, discrepancies also exist in the literature of this field, as another study showed that CAFs in a laryngeal human xenograft tumour model were not derived from the tumour cells as they had a mouse karyotype [105].

Independently of the origin of CAFs, the expression of EMT-TFs in CAFs seems to be required for their ability to promote cancer cells invasion and their expression is linked to poor prognosis. Snail expression in CAFs is necessary for their response to TGF-β, increased production of fibronectin and stiffness of the ECM. Moreover, co-culture of tumour cells with Snail-expressing CAFs enhanced the migratory capacity of the epithelial cells [106,107]. Infiltrating breast cancers in which CAFs express Snail have desmoplastic areas with anisotropic fibronectin and collagen fibres associated with poorer outcome of disease progression [106]. Twist1 expression in CAFs has also been associated with worst prognosis in gastric cancer and colorectal cancer where it promotes matrix stiffness by inducing the expression of paladin and collagen IV [108,109,110]. Furthermore, Zinc finger E-box-binding homeobox (ZEB)-1 and ZEB2 expression in CAFs from pancreatic ductal adenocarcinoma have been associated with poor prognosis and metastasis [111,112].

Finally, different studies have proved that CAFs isolated from different cancer types, invasive breast cancer or urinary bladder cancer, secrete TGF-β in their media, which can induce EMT in the adjacent cancer cells in a paracrine manner, by inducing the long non-coding RNA *ZEBNAT* and the *ZEB1* mRNA expression in the case of urinary bladder cancer [113,114]. TGF-β secreted by CAFs promotes breast cancer metastasis by inducing the expression of the lncRNA *HOTAIR* [115]. Moreover, TGF-β activity on CAFs increases the crosstalk between them and tumour cells; for instance, TGF-β enhances versican expression in CAFs which then upregulates the invasive capacity of ovarian cancer cells [116]. TGF-β also stimulates the secretion of interleukin (IL)-11 by CAFs, which then acts on colorectal cancer cells activating the gp130/STAT3 signalling pathway, which is required for tumour initiation but it is not important for the growth of tumours once established [117]. Interestingly, TGF-β downregulates caveolin expression in CAFS; at the same time, CAFs expressing low levels of caveolin show higher activation of the TGF-β signalling pathway, correlating with higher levels of phosphorylated SMAD2, which is translocated into the nucleus [118]. CAFS overexpressing TGF-β ligands, lose caveolin expression and undergo a metabolic reprogramming, shifting towards a catabolic metabolism, promoting mitophagy and autophagy, decreasing their mitochondrial activity and enhancing their aerobic glycolysis, which results in enhanced release of lactate [119]. Such metabolic reprogramming induced by TGF-β is associated with its capacity to downregulate the expression of isocitrate dehydrogenase 3α (IDH3α), which reduces the ratio of α-ketoglutarate to succinate and fumarate resulting in the stabilization of HIF1α and promotion of glycolysis, initiating the Warburg effect in CAFs [120]. Overexpression of IDH3α in CAFs abolishes the growth-promoting effect of CAFs in vivo.

## 6. TGF-β Signalling in Key Cell Types Involved in the Pathogenic Microenvironment

TGF-β not only exerts its functions in fibrosis by activating resident fibroblasts into myofibroblasts, as explained in the previous section. This cytokine also acts on other cell types present in the fibrotic and tumour microenvironment. In the case of epithelial cells, TGF-β can induce cell cycle arrest and apoptosis. Expression of activated TGF-β in transgenic mice induces epithelial apoptosis preceding the onset of lung fibrosis; fibrosis could be blocked by the use of a pan-caspase inhibitor [121]. Sustained apoptosis in the epithelial cell compartment is known to promote lung and liver fibrosis among other tissues [122,123]. Moreover, as explained above, TGF-β induces EMT to promote the differentiation of epithelial cells towards myofibroblasts or CAFS. 

Through a similar process, EndMT, TGF-β can induce endothelial cells (ECs) to adopt a mesenchymal phenotype. Via this transition, ECs can give rise to myofibroblasts expressing α-SMA, vimentin and collagens, while the expression of EC markers, such as vascular endothelial cadherin (VE cadherin), is reduced [124]. The endothelial origin of myofibroblasts has also been analysed in kidney and cardiac fibrosis using cell-fate mapping in mice [72,88,125]. TGF-β is responsible for the induction of EndMT in cardiac fibrosis as this phenomenon is decreased in Smad3^−/−^ mice [125] and in renal fibrosis, as partial deletion of TβRII reduces EndMT induction and fibrosis after UUO [126]. In different in vitro studies, it has been shown that TGF-β also requires the induction of EMT-TFs (Snail, Slug) to promote EndMT [127,128]. EndMT not only contributes to the myofibroblast and CAF pools of adjacent tissues but there is also evidence that is involved in angiogenesis. The expression of the EMT-TF Slug is for example necessary for angiogenic sprouting [129].

Macrophages are cells of the innate immune system, which are highly heterogeneous; they are involved in the primary response to microorganism infection, in inflammatory responses, homeostasis and tissue regeneration. However, macrophages can suffer a switch in their function and promote fibrosis by inducing myofibroblast activation and promoting tumorigenesis [130]. Depending on the stimuli that macrophages receive from their microenvironment, they are called M1, known as classical or pro-inflammatory, or M2, also known as alternative macrophages. The activation of M2 macrophages is promoted mainly by IL-4, IL-13, IL-10 and TGF-β; they secrete the same cytokines that activate them (IL-4, IL-13, IL-10 and TGF-β), they have high phagocytic capacity and produce ECM components, angiogenic and chemotactic factors. The release of TGF-β by macrophages perpetuates the myofibroblast activation, EMT and EndMT induction in the fibrotic tissue. M2 macrophages are essential for TGF-β-driven lung fibrosis, as macrophage depletion reduces fibrosis in a transgenic mouse with TGF-β1 specifically expressed in the lung [131]. Tumour associated macrophages (TAMs) have a similar secretory profile of that of M2 macrophages, they additionally release vascular epidermal growth factor (VEGF) and platelet-derived growth factor (PDGF), which are necessary for angiogenesis [132]. TGF-β secreted by TAMs induces EMT and acquisition of cancer stem cells characteristics in an in vitro model of HCC [133]; in lung cancer TAM-secreted TGF-β induces EMT in lung cancer cells by upregulating SOX9 expression via the c-Jun/Smad3 pathway [134]. TGF-β-induced expression of IRAK in TAMs has been proposed as a mechanism to negatively regulate TLR (Toll-Like Receptor) signalling and therefore suppress the cytotoxic activity of TAMs [135]. TGF-β also induces Snail expression in macrophages in order to induce their differentiation towards a TAMs phenotype, as silencing of Snail promotes M1 polarization and secretion of pro-inflammatory cytokines (TNF-α and IL12) [136]. Moreover, TGF-β reduces the infiltration of antigen-presenting dendritic cells into tumours and inhibition of TGF-β signalling by SB-431542, a potent chemical inhibitor of TβRI kinase, induces antigen presentation and T cell activation [137,138].

## 7. Stromal Derived TGF-β Modulates the Cancer Immune Response

The functions of TGF-β in cancer immunity have been widely investigated in the last decades. TGF-β exerts its anti-tumour activity by inhibiting the host tumour immunosurveillance. The balance between so-called “pro-tumour” and “anti-tumour” immunity is believed to influence the outcome of some solid cancers. TGF-β affects immune cells both directly and indirectly, in ways that inhibit anti-tumour effects, while promoting the escape from immunosurveillance of cancerous cells.

**Figure 1:**
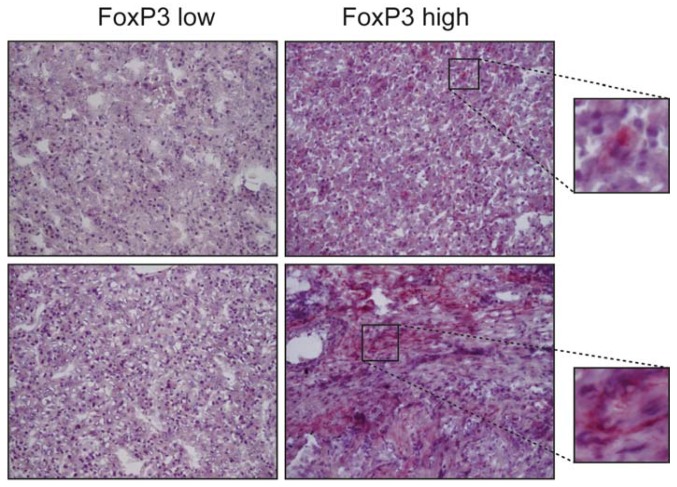
FoxP3 expression in 4 hepatocellular carcinoma (HCC) samples. FoxP3-positive cells belong principally to the CD4^+^ Treg subset, the presence of which is widely variable in different HCC tissues. The distribution pattern of FoxP3 does not show an apparent preference for any tumour sub-localization, as Tregs are found in parenchymal as well as stromal areas of HCC (upper right and lower right images, respectively). Representative images at 10×. The small images are a 200× of the selected area.

In particular, this cytokine inhibits the cytotoxic effects of CD8^+^ T and natural killer (NK) lymphocytes against cancer cells [139,140]. TGF-β signalling is responsible for blocking the generation of these tumour-specific cytotoxic T lymphocytes, which are capable of eradicating tumours [141]. In order to do so, TGF-β represses the cytotoxic gene program in T lymphocytes, including the expression of perforin, granzyme A and B, interferon-γ and FasL [140]. In part, TGF-β suppresses CD8^+^ T cell proliferation by promoting the interaction of Foxp1 with Smad2/3 to repress c-Myc expression, a major stimulator of T cell proliferation [142]; another mechanism is by interfering with IL2 production via inhibition of NFAT nuclear translocation [143,144]. Moreover, TGF-β polarizes the immune microenvironment committed against the cancer, through driving the differentiation of lymphocytes with a suppressive phenotype, like CD4^+^ regulatory T cells (Treg) [145,146]. Tregs are CD4^+^CD25^−^ T cells, which are converted into CD4^+^CD25^+^ T cells in response to TGF-β, a process that requires the induction of Foxp3 [147]. Tregs are involved in self-tolerance and immune suppression. They can suppress the proliferation of CD4 and CD8 cells in vitro and they are capable of also interfering with the production of IFN-γ by CD8 cells in vivo [148,149]. CD4^+^CD25^+^ Tregs suppress the cytotoxic activity of CD8^+^ T cells in a TGF-β dependent manner as lack of TβRII expression in CD8 cells renders them resistant to the effects of Tregs [150]. Tregs homed into the tumour microenvironment (Figure 1) secrete immunosuppressive cytokines, such as IL-10 and TGF-β itself, which may contribute to amplify the inhibitory effects of tumour-derived TGF-β on anti-tumour immunity effector cells.

Compatible with the complex role TGF-β plays in cancer progression, T cells that remain immunocompetent and succeed in limiting tumour growth have also been shown to require TGF-β signalling, which is thus classified as tumour suppressor signalling; indeed, T cells depend on their TGF-β/Smad4 signalling pathway in order to suppress gastrointestinal cancer, as demonstrated after genetic inactivation of TβRII in T cell progenitors [151].

CAFs represent another cell type that communicates with the immune system in the tumour microenvironment. Besides responding to TGF-β, CAFs are a source of this cytokine in desmoplastic solid cancers like HCC [152]. It is likely that CAF-derived TGF-β can impair the maturation of tumour dendritic cells (DCs), via blocking the expression of co-stimulatory proteins at the surface of these cells, which is necessary for the correct presentation of tumour-specific or associated antigens to reactive CD4^+^ T cells in secondary lymphoid tissues. TGF-β “educated” DCs drive the differentiation of CD4^+^ T cells to Treg, thus promoting immune tolerance to malignancy-related antigens [153].

All previous cases provide a strong demonstration of the multifunctionality of TGF-β, which is based on the fact that every cell type in a given tumour microenvironment can essentially respond to this cytokine. Such cell type-specific responses sometimes contribute to tumour suppression but the overwhelming evidence suggests that TGF-β is a pro-tumorigenic factor that instructs cells in the tumour microenvironment to assist the growing tumour so that cancer eventually wins over the homeostatic control evolved by adult vertebrate tissues.

## 8. TGF-β as a Link between Fibrosis and Hepatocellular Carcinoma Progression

**Figure 2:**
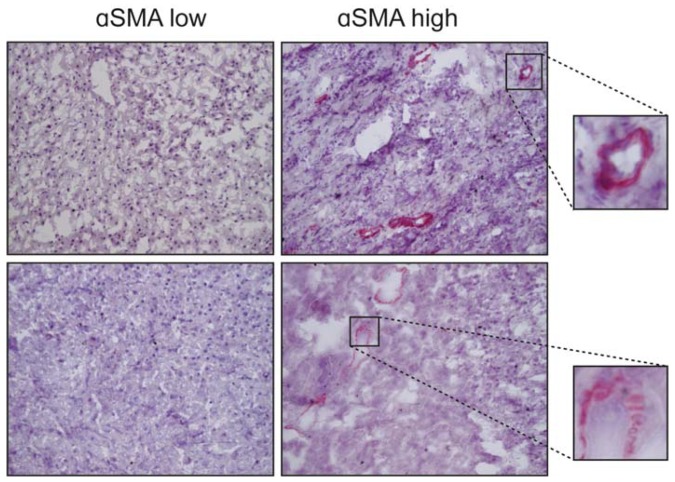
Staining of α-smooth muscle actin (αSMA) in 4 HCC samples. The differentiated myofibroblasts within the tumour are the main cells expressing this actin isoform and are located in the stromal septa or around the blood vessels. Representative images at 10×. The small images are a 200× of the selected area.

The fibrotic stage of liver disease is a major risk factor for the onset of HCC [154]. Depending on the causative aetiology (viral infection, alcoholism or other), cirrhosis can induce various patterns of activating or inactivating mutations which target genes of major pathways, such as *TP53*, *CTNNB1*, *AXIN1* and *PTEN*, thereby supporting the onset and progression of HCC [155]. Therefore, elucidating the molecular and cellular mechanisms which underlie the fibrotic process might promote the discovery of therapeutic interventions that may slow down or even reverse the progression of cirrhosis, thus reducing the related risk of HCC [156]. TGF-β plays a major fibrogenic role through both direct and indirect mechanisms. As explained earlier in this article, TGF-β directly stimulates target cells to synthesize and deliver extracellular matrix molecules such as collagens, fibronectin and laminins within the stromal milieu [46]. In fibrotic liver, TGF-β can be released by different cell types, including hepatitis C virus (HCV)-infected hepatocytes, HCV-exposed resident macrophages (Kupffer cells) or Tregs [157,158]. TGF-β triggers fibrogenic signalling in HSCs, which is enhanced in the presence of bowel-derived bacterial compounds, such as lipopolysaccharide [159]. In the liver, this cytokine induces the differentiation of contractile cells called myofibroblasts. The origin of these cells is controversial, as discussed in the previous section, as they have been proposed to derive from many different likely precursors, including epithelial cells via the EMT, inflammatory macrophages and HSCs [160,161,162,163]. Myofibroblasts are present in several anatomic sites and are involved in multiple physiologic and pathologic processes, primarily in the regulation of tissue morphology [164], wound healing [165] and fibrosis [162]. The most common marker used for identifying myofibroblasts is αSMA, although this actin isoform is not exclusive to these cells but is also expressed in pericytes, bone marrow mesenchymal and hematopoietic stem cells [164]. αSMA is abundantly present in hepatic cirrhosis as well as in HCC (Figure 2). Sasaki et al., have recently demonstrated that HCV-exposed Kupffer cells can activate HSCs through the release of the chemokine CCL5, which in turn increases the expression of inflammasome-related genes (IL-1β, IL-6, CCL5 itself) and fibrosis markers, such as TGF-β1, collagens, MMP2 metalloproteinase and αSMA, in HSCs [157].

Besides promoting fibrosis, TGF-β can also inhibit its reversion through a number of mechanisms. NK cells have been involved in the resolution of fibrosis, by inducing apoptosis in HSCs [166]. However, some researchers have found that in late stage cirrhotic liver, HSCs can induce the clearance of NK cells through a mechanism called emperipolesis [167]. This process consists in the formation of a cell-in-cell structure achieved by the engulfment of NK cells by HSCs, followed by the induction of apoptosis in NK cells by TGF-β produced by HSCs [168]. In a murine model of fibrosis, a particular subset of recruited liver macrophages, called Ly-6C(lo), has been reported to be responsible for resolving fibrosis induced by CCl_4_, by secreting metalloproteinases (MMP-9 and -12). The expression of TGF-β and Thrombospondin-1, the activator of latent TGF-β, was downregulated in Ly-6C(low) macrophages [169]. Mesenchymal stem cells have recently been proposed as negative regulators of HSCs-induced fibrogenesis, through the secretion of a protein known as milk fat globule-EGF factor 8 (MFGE8), which binds to α_v_β_3_ integrin of HSCs, thus leading to the downregulation of TGF-β receptor I and the consequent inhibition of pro-fibrotic pathways in HSCs [170]. 

**Figure 3:**
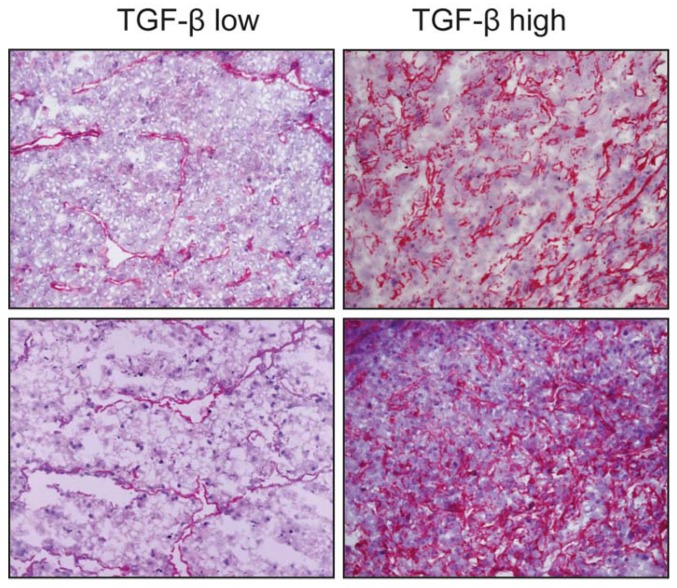
Expression of transforming growth factor-β (TGF-β) in 4 HCC samples. Overall staining is evident, with a preferential sub-endothelial localization, possibly perisinusoidal, especially in tissues with lower expression. The cytokine delivery sites appear to be closely associated to parenchymal cells in HCC nodules. Representative images at 10×.

TGF-β is often highly expressed in HCC tissues (Figure 3). After the onset of HCC, TGF-β supports tumour progression by profoundly reshaping the phenotype of several target cells in the tumour. It induces stemness genes, promotes the EMT and enhances the motility of cancerous hepatocytes, while increasing their responsiveness to growth factors such as EGF [171,172,173]. TGF-β stimulation induces HCC cells to release a plethora of soluble molecules that, in turn, target stromal cells, often converting them into tumour accomplices. Connective tissue growth factor (CTGF), for example, is secreted by TGF-β-exposed HCC cells and enhances CAF proliferation, thus increasing their support of tumour growth and dissemination in a murine xenograft model of HCC [174]. Compared to peritumor fibroblasts, CAFs secrete high levels of certain chemokines, such as CCL2, CCL5, CCL7 and CXCL16. CCL2 and CCL5 stimulate the hedgehog (Hh) pathway to increase the migration but not the invasion of HCC cells. By contrast, CCL7 and CXCL16 activate both processes through the enhancement of the TGF-β pathway in these cells [175]. 

## 9. Systemic Therapy Based on TGF-β Pathway Inhibition

The drug-based therapeutic approach for patients with HCC is currently a top field of interest for clinicians and researchers, because of the urgent need to respond to the increasing number of patients who are not eligible for surgery or loco-regional therapies because they already have a more advanced stage of disease. Sorafenib and Regorafenib are, to date, the only approved first and second line treatments, respectively, in patients with HCC [176]. Nevertheless, personalized therapy based on biological characteristics of patients aiming at improving therapeutic effectiveness or to avoid drug resistance [177] is still lacking.

The TGF-β pathway is considered a hallmark of HCC, nevertheless it is expressed at different levels both in liver cell types (Figure 3) and in the serum of HCC patients and does not correlate with the status of the disease according to the Barcelona clinic liver cancer (BCLC) staging system. This stresses the importance of molecular studies in order to stratify patients and identify those more likely to benefit from different drugs like TGF-β inhibitors [178]. Therefore, TGF-β has recently been proposed as an interesting druggable target in a number of experimental preclinical models [179]. Various new biomarkers, such as PMEPAI and SKIL, have been identified and tested in preclinical ex vivo models in which HCC specimens have been treated with TGF-β under “in vitro” conditions [180]. A pilot study conducted on human HCC tissues also reported a precision medicine strategy for identifying responder and non-responder tissues to galunisertib on the basis of molecular diagnostic patterns [181]. Galunisertib is a clinically tested low molecular weight inhibitor of the TβRI kinase. In this scenario the microenvironment is considered to play a key role, since the different molecular and cellular composition of the milieu, including the activation of HSCs (Figure 2), is responsible for a more aggressive phenotype. Considering that TGF-β is a fundamental pathway orchestrating the microenvironment and affecting immune inflammatory responses such as the Treg cells (Figure 1), linking modern immunotherapy trials to TGF-β signalling-related biomarkers is reasonable. The expression and role of the PD-1/PD-L1 mediated response are not fully understood but they represent the scientific rationale for targeting immune check points with anti PD-1 inhibitors in further clinical investigations [182]. A role for TGF-β in this pharmacological regime deserves further investigation. Classifying patients in order to predict the clinical outcome and the overall survival is crucial to define a therapeutic road map for each patient. Currently none of the classifications [183,184,185], including the BCLC system, are realistically adapted to daily-life practice. Recently, in a prospective study, a five-gene signature was reported to predict the survival of the patients independently of the therapy they will receive, suggesting that the molecular characteristics of the tumour is what drives the prognosis [186]. This exciting new finding requires further validation but if confirmed, will offer strong evidence supporting the personalized therapy approach.

## 10. Concluding Remarks

**Figure 4:**
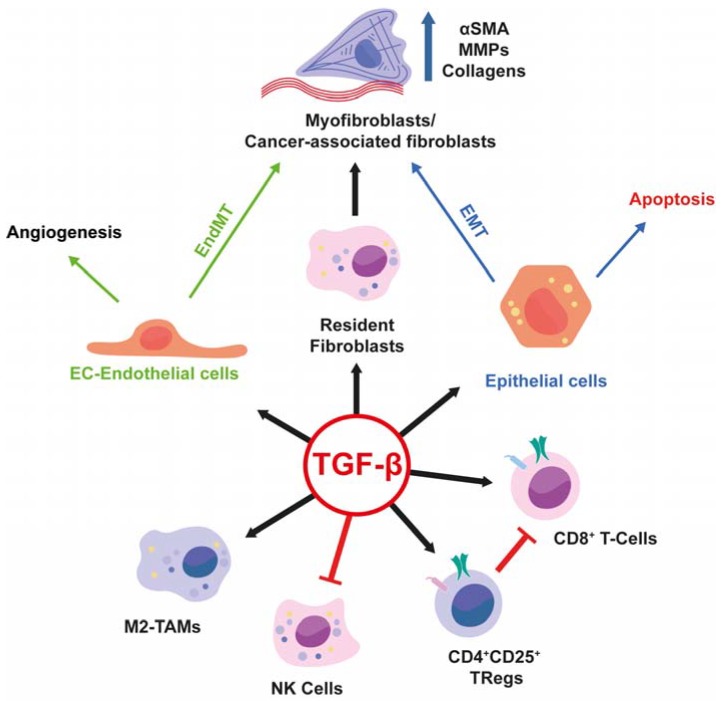
Graphical abstract highlighting the pleiotropic actions of TGF-β on different cell types, which explain how TGF-β influences the microenvironment, contributing to the development and progression of fibrosis and cancer. Red lines indicate inhibition of the indicated cell type activity. Black arrows indicate activation of the indicated cell type. Green arrows indicate that Endothelial cells upon TGF-β stimulation can undergo either Angiogenesis or EndMT. Blue Arrows indicate that epithelial cells after TGF-β stimulation can undergo apoptosis or EMT.

The biology of fibrotic or tumour tissues is often perplexing. Yet, much detail in understanding this complexity has been achieved over the past decades. Focus on the cytokine TGF-β in the context of fibrosis and cancer progression is very useful; this is because the elucidation of mechanisms that regulate TGF-β ligand deposition and activation in the ECM, signalling receptors and intracellular mediators and expression of target genes of this pathway offer a truly integrative perspective in analysing communications and reactions between all cell types that participate in the progression of these pathologies in every human organ. The article has highlighted important key mechanisms that exemplify the actions of TGF-β in such pathologies and selected illustrative cellular and molecular interactions to guide the reader through the evolution of the pathologic tissue microenvironment (Figure 4). This approach emphasizes the logic behind the use of TGF-β chemical inhibitors as a new line of defence against fibrotic disorders or cancer and a cluster of biomolecules that are regulated by TGF-β signalling, as useful biomarkers of disease progression and therapeutic efficacy.
